# Effect of Oral Screen Training After Stroke—A Randomised Controlled Trial

**DOI:** 10.1111/ger.12803

**Published:** 2024-12-05

**Authors:** Jesper Dalum, Pia Skott, Elisabet Åkesson, Emmelie Persson, Åsa Karlsson, Henrike Häbel, Åke Seiger, Anita McAllister, Kerstin Johansson, Gunilla Sandborgh‐Englund

**Affiliations:** ^1^ Academic Center for Geriatric Dentistry Stockholm Sweden; ^2^ Department of Dental Medicine Karolinska Institutet Stockholm Sweden; ^3^ Folktandvården Stockholm AB, Public Dental Services Stockholm Sweden; ^4^ Division of Neurogeriatrics, Department of Neurobiology Care Sciences and Society, Karolinska Institutet Stockholm Sweden; ^5^ R&D Unit, Stockholms Sjukhem Stockholm Sweden; ^6^ Medical Statistics Unit, Department of Learning, Informatics, Management and Ethics Karolinska Institutet Stockholm Sweden; ^7^ Aleris Rehab Station Stockholm Sweden; ^8^ Division of Clinical Geriatrics, Department of Neurobiology, Care Sciences and Society Karolinska Institutet Stockholm Sweden; ^9^ Division of Speech Language Pathology, Department of Clinical Science, Intervention and Technology Karolinska Institutet Stockholm Sweden; ^10^ Women's Health and Allied Health Professionals Theme, Medical Unit Speech and Language Pathology Karolinska University Hospital Stockholm Sweden

**Keywords:** dysphagia, multidisciplinary approach, oral screen, orofacial training, RCT, rehabilitation, stroke

## Abstract

**Objective:**

To assess the effects of oral screen training in patients with dysphagia post‐stroke.

**Background:**

Oral screen training has been identified as an effective method for improving orofacial and oropharyngeal motor functions. However, the evidence supporting a positive transfer effect on swallowing capacity post‐primary stroke rehabilitation is still unclear. The aim of this randomised controlled trial was to investigate the effect of a 12‐week oral screen training programme using a prefabricated oral screen, with swallowing capacity as the primary outcome.

**Materials and Methods:**

In a randomised trial, stroke survivors with residual dysphagia post‐rehabilitation were randomised into intervention group (*n* = 12) and control group (*n* = 12). The intervention group underwent 12 weeks of oral screen training. The main outcome was swallowing capacity, with lip force as a training indicator. Secondary outcomes were assessed by the Eating Assessment Tool, Masticatory performance, Nordic Orofacial Test—Screening, Life Satisfaction Questionnaire and the Edmonton Symptom Assessment System.

**Results:**

At the 3‐month follow‐up, the group that trained with an oral screen showed a significantly greater increase in lip force than the control group (mean lip force increase 10.2 N vs. 3.1 N; *p* = 0.02). There was no significant improvement in swallowing capacity (mean increase 0.7 mL/min vs. 0.8 mL/min; *p* = 0.43), or in any of the secondary variables in the intervention group relative to the control group.

**Conclusion:**

The findings from this study showed that oral screen training initiated after completion of regular rehabilitation post‐stroke can increase lip force. However, there was no indication of any transfer effect on swallowing capacity.

**Trial Registration:**

Clinicaltrial.gov identifier: NCT03167892

## 1 Introduction

Oropharyngeal dysphagia is a common sequela after stroke, impairing an individual's capacity for safe and efficient nourishment and hydration [1, 2]. Compromised swallowing function sets the stage for grave health risks, including aspiration pneumonia, malnutrition, dehydration and a deterioration in quality of life [3]. Further complicating matters, impaired orofacial control frequently results in the involuntary leakage of saliva, liquids and food, primarily due to reduced lip strength, thus adversely influencing oral health‐related quality of life [4]. It is consequently of clinical relevance to find effective post‐stroke rehabilitation strategies aimed at restoring oropharyngeal and orofacial function, including mobility and strength in lips and face.

Oral screen training has been identified as an effective method for improving orofacial functions and oropharyngeal swallowing function [4, 5, 6, 7]. The methodology, physiology and outcomes of oral screen training are based on the interdependence of the orofacial, pharyngeal and suprahyoid and infrahyoid muscles. Targeted strengthening of the buccopharyngeal musculature, such as through oral screen training, may improve hyoid bone elevation during swallowing, thereby bolstering the defence of the larynx and airways and enhancing the efficiency of pharyngeal bolus transport [8]. Furthermore, oral screen training may also extend its benefits to the oral phase by improving lip seal and clearing the oral cavity [4].

However, while the improved lip force and swallowing capacity from oral screen training shows promise, the evidence supporting its positive transfer effect on swallowing capacity post‐primary rehabilitation is still unclear. Previous studies have focused on oral screen training during primary rehabilitation within the first 3 months post‐stroke [5, 6]. In contrast, the present study focuses on outpatients still suffering from dysphagia after completing primary stroke rehabilitation to evaluate the effect of a training procedure after spontaneous recovery post‐stroke had ceased [9]. The aim of this randomised controlled trial was to evaluate the effects of oral screen training in patients with residual dysphagia post regular primary rehabilitation, and to test if a 12‐week oral screen training programme for patients with post‐stroke dysphagia improves their swallowing capacity.

## 2 Materials and Methods

### 2.1 Study Design

The study was conducted as a prospective, open label, randomised and controlled clinical study at the Academic Center for Geriatric Dentistry, Stockholm, Sweden. Informed written and verbal consent was obtained from all participants prior to inclusion. The study was performed according to the Helsinki Declaration, approved by the Swedish Ethical Review Authority (Dnr 2016/2592‐31/1 and amendments 2018/867‐32, 2019‐02387, 2020‐01278, 2021‐00214).

### 2.2 Participation and Procedure

The study included patients with persistent subjective and/or objective swallowing difficulties after stroke rehabilitation. The intervention group was instructed to undergo oral screen training for 12 weeks, while the control group received no training.

**FIGURE 1:**
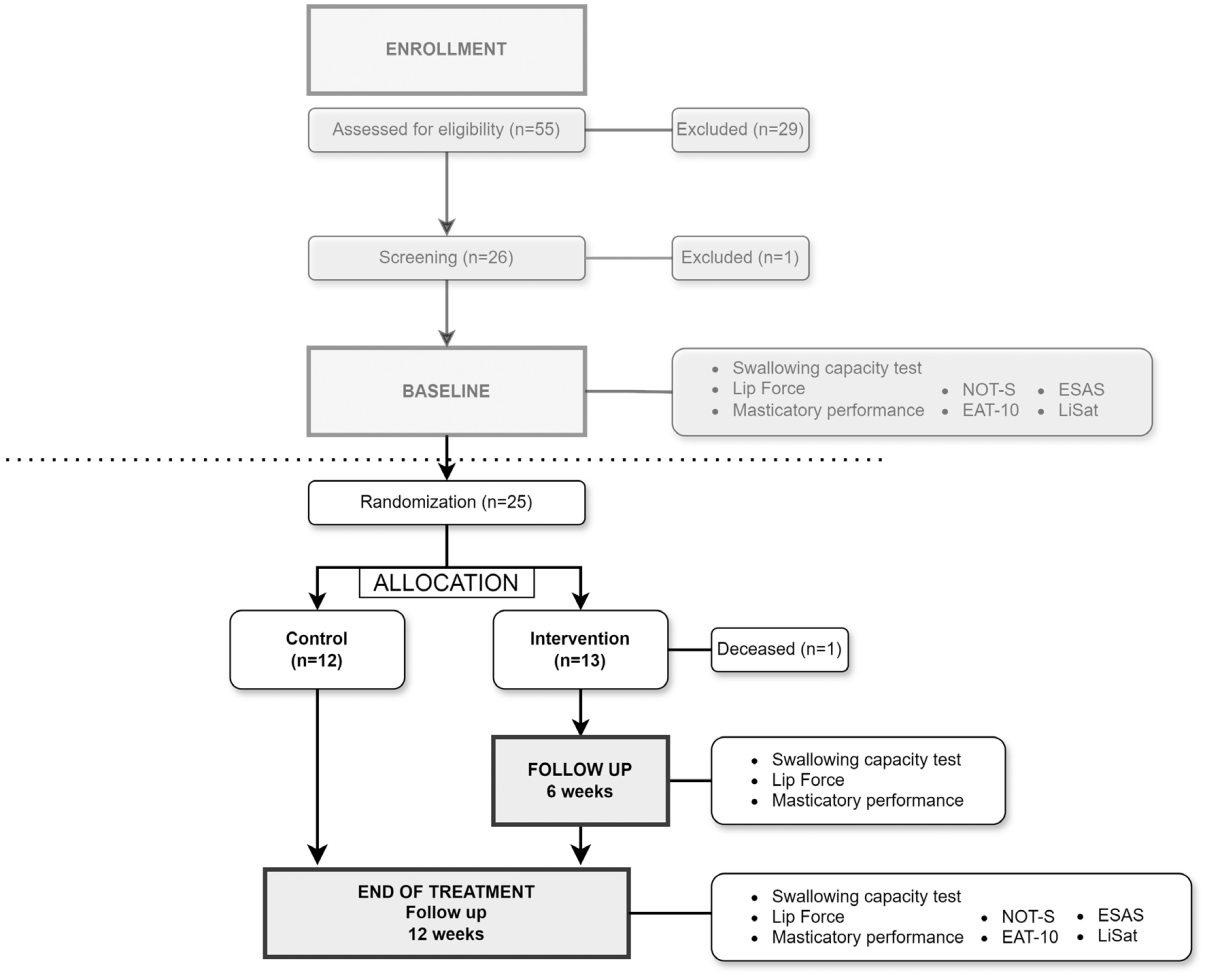
Flow chart of the study from enrolment to end of treatment, including measurements at Baseline, 6‐week follow‐up and end of treatment (12‐week follow‐up). ESAS, Edmonton Symptom Assessment System [20]; EAT‐10, Eating Assessment Tool [15]; LiSat, Life Satisfaction Questionnaire [19]; NOT‐S, Nordic Orofacial Test [18].

A total of 11 stroke rehabilitation clinics participated in the recruitment process. Figure 1 shows a flow chart of participants included in the study and the measurements included. Eligible participants were identified and informed about the study at discharge from post‐stroke primary rehabilitation via speech and language pathologists in outpatient clinics. These persons were contacted and invited to participate in the screening 3–6 months after discharge. At the first meeting, consent forms were collected, and a screening process ensured that participants met the inclusion criteria. The criteria included being ≥ 50 years of age, having been diagnosed with a first or second stroke with remaining subjective and/or objective swallowing difficulties after completing primary rehabilitation ≥ 3 months ago and having natural teeth corresponding to category A1–A3 by the Eichner Index [10]. Participants with remaining moderate to severe impressive aphasia, cognitive impairment or unilateral neglect were excluded from the study.

A full description of the screening process including methods, cut‐offs and an analysis of baseline data has been previously published [11] and summarised below. At baseline, the swallowing capacity, lip force, masticatory performance and patient‐related outcome measures were evaluated. After the baseline registrations, the participants were randomised to either the intervention group (oral screen training for 12 weeks) or to the control group. The randomisation was performed in blocks of eight (4 control/4 intervention). The randomisation was constructed by an external party, Karolinska Trial Alliance and was stored in sealed envelopes at the dental clinic at Stockholms Sjukhem, Stockholm, Sweden. The participants in the intervention group received an oral screen (IQoro, MYoroface AB, Hudiksvall, Sweden). They were instructed to place the oral screen behind closed lips (and in front of the teeth), while keeping their head upright, and to pull the screen distinctly forward for 30 s. During each pull, the lips should be firmly pressed together to keep the oral screen in place, while applying as much force as possible. The procedure was to be repeated three times, with a 3‐s rest between each pull. Oral and written instructions were provided by a speech and language pathologist. The participants in the intervention group were instructed to train three times a day and to keep a training diary. Follow‐up assessments were conducted after 6 weeks for the intervention group, and at 12 weeks for both groups.

### 2.3 Outcome Measurements

Swallowing capacity was the main outcome, assessed with a swallowing capacity test [12]. Based on prior study findings [13], a sample size calculation determined that 54 participants would be needed to detect a 40% higher proportion of the participants in the intervention group reaching 10 mL/s in the SCT after the training programme, with 80% power and a 5% significance level. Lip force was evaluated as an indicator of performed training and assessed using an oral screen connected to a handheld digital force gauge [14]. Secondary outcomes were assessed using several tools such as the Eating Assessment Tool (EAT‐10), for self‐reported eating and swallowing difficulties [15], masticatory performance, measured by the mixing ability of two‐coloured wax [16, 17], the Nordic Orofacial Test—Screening (NOT‐S) [18] for subjective and objective orofacial function, the Life Satisfaction Questionnaire (LiSat‐11) [19] and the Edmonton Symptom Assessment System (ESAS) [20] for life satisfaction and subjective symptoms.

### 2.4 Statistical Methods

Descriptive statistics, presenting mean and standard deviation (SD) for continuous variables or median and interquartile range (IQR) for categorical variables, were used to describe the distribution of group outcomes. We used linear regression and ordered logistic regression to analyse the association between the intervention and the outcome variables. We performed analyses of longitudinal effects on lip force within the intervention group using mixed effects regression models. Two‐sided *p*‐values are presented and a *p*‐value below 0.05 was considered significant. The analyses were performed using STATA version 16.1 (StataCorp, College Station, TX, USA).

## 3 Results

In total, 25 participants were included in the study. One participant in the intervention group was deceased after baseline, resulting in 24 participants completing the study protocol. The intervention and control groups did not differ significantly with regard to age at diagnosis (median 76 and 77 years, respectively; *p* = 0.55) and sex (five and six females, respectively; *p* = 0.56); see Table 1.

**TABLE 1:** Descriptive statistics of the control and intervention group.

Variables	Control (*n* = 12)	Intervention (*n* = 12)
Sex		
Female	6	5
Male	6	7
Age median (min–max)	77 (61–85)	76 (56–85)
Stroke to baseline median months (min–max)	7 (4–12)	8 (3–12)

Data on swallowing capacity, lip force, NOT‐S, EAT‐10, masticatory performance, LiSat and ESAS at baseline and end of study at 3 months are presented in Table 2.

**TABLE 2:** Swallowing rate, lip force, NOT‐S, EAT‐10, masticatory performance, LiSat and ESAS at baseline and end of study at 3 months.

Variables	Intervention (*n* = 12)	Within group diff	Control (*n* = 13)	Within group diff	Between group diff
	Mean or *Median*	SD (IQR)	Mean or *Median*	SD (IQR)	*p*
Swallowing rate (mL/s)					
Baseline	4.3	3.8	9.0	5.9	
Follow‐up (3 months)	5.0	3.7	9.8	6.0	0.433^a^
Lip force (*N*)					
Baseline	20.4	8.0	19.9	11	
Follow‐up (3 months)	30.6	11.2	23.0	10.5	**0.020** ^a^
NOT‐S					
Baseline	4.0	(3)	5.0	(1.8)	
Follow‐up (3 months)	3.0	(1.5)	4.5	(2.3)	0.415^b^
EAT‐10					
Baseline	12.2	9.9	9.7	8.5	
Follow‐up (3 months)	8.2	8.7	8.6	7.6	0.349^a^
Masticatory performance					
Baseline	2.0	(0)	2.0	(1.3)	
Follow‐up (3 months)	2.0	(0.3)	2.0	(0)	0.901^b^
LiSat					
Baseline	43.5	10.9	39.7	7.8	
Follow‐up (3 months)	42.7	9.1	42.3	8.2	0.174^a^
ESAS					
Baseline	25.3	18.1	28.3	12.4	
Follow‐up (3 months)	25.3	13.8	24.4	12.2	0.303^a^

*Note:* Statistically significant (*p*‐value  < 0.05) in bold.

Abbreviations: EAT‐10, Eating Assessment Tool [15]; ESAS, Edmonton Symptom Assessment System [20]; LiSat, Life Satisfaction Questionnaire [19]; NOT‐S, Nordic Orofacial Test [18].

^a^ Linear regression.

^b^ Ordered logistic regression.

At baseline, the swallowing capacity was significantly higher in the control group than in the intervention group (mean 9.0 and 4.3 mL/s; *p* = 0.043). The intervention group and control group did not differ significantly in any other baseline measurements. At the end of the study, there was a small and non‐significant increase in swallowing capacity in both the groups. The swallowing capacity of the control group remained higher than the intervention group at follow‐up (9.8 and 5.0 mL/s, respectively; *p* = 0.037). Between groups, there was no significant change in swallowing capacity (*p* = 0.433), indicating no association between change in swallowing capacity and the oral screen training. At the 6‐week and 12‐week follow‐ups, the intervention group showed a significant increase in lip force from baseline. Increased lip force at 12 weeks was associated with the intervention (*p* = 0.020). For the other secondary outcomes tested, NOT‐S, EAT‐10, masticatory performance, LiSat and ESAS, there was no clinically relevant difference compared to baseline nor with the intervention. Eight out of 12 participants submitted their training diary at the 12‐week follow‐up. Among these, the average number of weekly exercises was 17 out of a maximum 21 (three times/day).

## 4 Discussion

This study's main objective was to evaluate the effect of oral screen training on swallowing capacity in participants suffering from dysphagia after primary stroke rehabilitation. The findings show adherence by the intervention group that trained according to the study protocol and demonstrated a significant improvement of lip force at the end of treatment. Although the training improved lip force, there was no significant difference as regards swallowing capacity between baseline and end of treatment. In addition, there was no observed significant transfer effect of the oral screen training on the secondary outcomes.

Lip strength training can be expected to increase lip strength in patients after stroke and in patients with lip incompetence [4, 21]. We observed a significant increase in lip force already at the 6‐week follow‐up, which is in line with previous studies reporting similar improvements after 4–5 weeks of oral screen training [4, 6]. Hägglund et al. [6] noted an increase in swallowing capacity after 5 weeks in both the control and intervention groups. We did not observe any significant improvement between baseline and the 12‐week follow‐up concerning the swallowing capacity in either group; instead, the observed baseline difference between groups remained unchanged. This inconsistency between the present study with previous findings could be due to dissimilar inclusion criteria and/or the use of a different oral screen. Also, Hägglund et al. [6] initiated the training approximately 4 weeks post‐stroke, while our training was initiated post initial rehabilitation phase.

### 4.1 Limitations

The study aimed to recruit stroke patients discharged from primary rehabilitation who had persistent subjective and/or objective swallowing dysfunction. However, the outbreak of the SARS‐CoV‐2 pandemic and the societal restrictions implemented to protect the elderly and individuals with chronic disorders significantly hindered participant inclusion as planned. Due to recruitment difficulties, we expanded enrolment from one stroke rehabilitation clinic to 11 clinics. The inclusion criteria were also modified: the age requirement was lowered from ≥ 65 to ≥ 50 years, and the planned inclusion criteria of 8–12 months post‐stroke was redefined as > 3 months post primary rehabilitation. As previously reported in the baseline study, recruitment barriers included difficulties in identifying, contacting and motivating potential participants within standard care pathways. We also found that barriers to patient participation in clinical trials can be underestimated, that is, by factors such as physical limitations, post‐stroke depression and reduced healthcare contact in the post‐rehabilitation phase [11]. Despite a prolonged recruitment period, the study still only reached a limited sample size. Regarding compliance, eight out of 12 participants returned their training log at the 12‐week follow‐up. To fulfil the training programme including oral screen training three times per day, every day for 12 weeks was demanding, as observed in the training logs revealing missed training sessions. However, at the follow‐ups, the patients reported a strong motivation to continue. It was found that the lip force in the intervention group improved at both the 6‐ and 12‐week follow‐ups, suggesting that participants trained to the best of their ability.

### 4.2 Subject for Future Studies

Inadequate sealing of the lips may lead to deterioration of the oral environment, mainly due to saliva leakage and reduced clearance of the oral cavity. Future intervention studies of oropharyngeal dysphagia are suggested to also include objective and subjective measures of saliva function and reporting on oral health‐related quality of life. In addition, the barriers for post‐stroke patient participation in clinical trials have been suggested to be underestimated, and it is therefore of importance to explore the patients' own views on participation in clinical studies.

### 4.3 Multidisciplinary Approach for Person‐Centred Care

Our previously published baseline study demonstrated that the participants formed a diverse group reflecting a broad population, in terms of post‐stroke orofacial dysfunction [11]. Interestingly, participants generally reported high levels of life satisfaction, despite the presence of objectively measured dysfunctions both at the start and end of the study, suggesting that the study participants have developed effective coping strategies.

Considering the complexity of dysphagia, the multidisciplinary approach to investigate the effects of oral screen training post‐stroke was beneficial, with competencies within the research team being dynamic and complementary. The study design also considered the Swedish dental care subsidy, which is activated for stroke patients with residual functional disabilities 6 months post‐stroke. In Sweden, the standardised patient‐centred care processes post‐stroke include essential checkpoints for oral health risk assessment and standardised information regarding the dental care subsidies at discharge. The participants' diversity underscores the need for a multidisciplinary approach in future research. It also highlights the importance of collaborative, inter‐professional efforts in providing comprehensive and individualised rehabilitation and care for stroke survivors, addressing multiple perspectives. Following discharge, dental professionals can play a key role in the rehabilitation of post‐stroke survivors by identifying dysphagia symptoms during regular check‐ups, thereby facilitating referrals to primary care.

## 5 Conclusion

The findings from this study show that oral screen training initiated after completion of regular rehabilitation post‐stroke after 3 months post‐stroke diagnosis can increase lip force. However, there was no indication of any transfer effect on the swallowing capacity. The results should be interpreted with consideration to the limited sample size.

## Author Contributions

J.D., P.S., E.Å., Å.S., A.M. and G.S‐E. designed the study. Å.K. and E.P. screened the patients for eligibility. Å.K., E.P. and J.D. collected the data. J.D., P.S., E.Å., K.J., A.M., H.H. and G.S‐E. analysed the data. J.D., P.S., E.Å., K.J., Å.S., A.M. and G.S‐E. wrote the manuscript. All authors reviewed the manuscript.

## Ethics Statement

This study was performed according to the Helsinki Declaration, approved by the Swedish Ethical Review Authority (Dnr 2016/2592‐31/1 and amendments 2018/867‐32, 2019‐02387, 2020‐01278, 2021‐00214).

## Conflicts of Interest

The authors declare no conflicts of interest.

## Data Availability

The data that support the findings of this study are available upon reasonable request.
